# Modeling for Single-Photon Avalanche Diodes: State-of-the-Art and Research Challenges

**DOI:** 10.3390/s23073412

**Published:** 2023-03-24

**Authors:** Xuanyu Qian, Wei Jiang, Ahmed Elsharabasy, M. Jamal Deen

**Affiliations:** 1 Department of Electrical and Computer Engineering, McMaster University, Hamilton, ON L8S 4K1, Canada; 2 Department of Electrical Engineering, Toronto Metropolitan University—Cairo, New Administrative Capital, Egypt; 3 School of Biomedical Engineering, McMaster University, Hamilton, ON L8S 4L8, Canada

**Keywords:** single-photon avalanche diodes (SPADs), imaging system, complementary metal-oxide-semiconductor (CMOS) photodiodes, photon detection probability (PDP), dark count rate (DCR), afterpulsing probability (AP), silicon photomultiplier (SiPM), timing jitter

## Abstract

With the growing importance of single-photon-counting (SPC) techniques, researchers are now designing high-performance systems based on single-photon avalanche diodes (SPADs). SPADs with high performances and low cost allow the popularity of SPC-based systems for medical and industrial applications. However, few efforts were put into the design optimization of SPADs due to limited calibrated models of the SPAD itself and its related circuits. This paper provides a perspective on improving SPAD-based system design by reviewing the development of SPAD models. First, important SPAD principles such as photon detection probability (PDP), dark count rate (DCR), afterpulsing probability (AP), and timing jitter (TJ) are discussed. Then a comprehensive discussion of various SPAD models focusing on each of the parameters is provided. Finally, important research challenges regarding the development of more advanced SPAD models are summarized, followed by the outlook for the future development of SPAD models and emerging SPAD modeling methods.

## 1. Introduction

Single-photon avalanche diodes (SPADs) play a crucial role in a variety of imaging systems due to their high sensitivity allowing single-photon detection. Compared to other types of optical detectors, such as photomultiplier tubes (PMTs), SPADs or SPAD arrays are compatible with magnetic fields, have high timing resolution, and can operate under lower voltages. Therefore, SPADs are widely used in fluorescence lifetime imaging (FLIM), time-of-flight positron emission tomography (ToF-PET), and Raman spectroscopy (RS) [1,2] for biomedical imaging applications and in light detection and ranging (LiDAR) systems for autonomous driving. With the detection ability down to a single photon, SPADs can detect very low-intensity fluorescence decay in FLIM. In ToF-PET applications, SPADs can be integrated into arrays with time-to-digital converters (TDCs) to form silicon photomultipliers (SiPMs) or digital silicon photomultipliers (dSiPMs) to further improve the timing resolution and increase the compactness of PET systems. In LiDAR applications, SPADs can be integrated into arrays for higher throughput and improved noise performance [3,4,5,6,7,8]. With the advancement of silicon manufacturing technologies, many SPADs are fabricated using various CMOS technologies, from standard technologies to custom technologies, depending on the specific applications. With CMOS technologies, SPADs can be easily integrated with different readout circuits for following-stage signal processing, such as active quench and reset circuits, time-gated circuits, analog counters, and TDCs [9,10]. All these advantages make SPADs better candidates than conventional PMTs and APDs in optical sensing applications. A good summary of CMOS-based SPADs was presented in [1]. SPADs fabricated using specific CMOS technologies may suffer from reduced photon detection probability (PDP) due to passivation layers above the device and the thin depletion region of the junctions. They may also suffer from high dark count rates (DCR) due to the increased doping concentration in advanced CMOS technology nodes [1]. Therefore, SPAD models are important to simulate and predict performance before fabrication. However, few publications focusing on the SPAD model development have been presented. To our best knowledge, this paper is the first comprehensive review of SPAD models. We have discussed the progress of SPAD models, ranging from the conventional multiplication models in the 1960s to two-dimensional SPAD models published in recent years [11,12,13,14]. In this paper, comparisons of different SPAD models are presented, the modeling process and methods are discussed, and the future directions of SPAD models are described. Circuit designers may efficiently improve their designs by obtaining a clearer idea of how to improve their own SPAD models from this paper.

Many researchers have contributed to the development of SPAD modeling from different perspectives. For good SPAD models, basic operational principles and technology-related parameters should be considered concurrently. For SPADs, two major types of models, physical models and circuit models will be discussed in this paper. More specifically, physical SPAD models are developed to simulate the physical mechanisms of an individual device. Such mechanisms are usually related to intrinsic properties of the material, structures, and operating principles of the device. SPAD circuit models are proposed to describe SPADs’ behavior in real circuits. Without either one of these two types of models, the actual performance of SPAD-based detection systems cannot be accurately predicted, which makes the design and optimization of SPADs more challenging.

It is known that the performance of SPADs is strongly related to specific fabrication technologies. For example, different fabrication technologies usually have different defect levels, thus causing undesired differences in DCR and PDP between simulations and measurements [15]. In fact, technology-related parameters affect almost all other important performances of SPADs, such as AP and timing jitter [16,17,18].

For PDP models, the most considered aspects are light absorption and avalanche triggering. The material, SPAD structures, doping concentration, and optical properties of any layers of the device can significantly affect SPADs’ PDP, making the modeling process more complicated. Some of the above-mentioned parameters are confidential and not accessible to users. Therefore, estimated values for some parameters are used in the model development, which may cause large differences between simulations and measurements.

For DCR models, dark carriers have replaced photon-generated carriers as the research focus. These carriers generated under total dark conditions can initiate undesirable avalanche events when the electrical field is high enough. The generation of dark carriers in SPADs is due to two major mechanisms: thermal generation and tunneling [19,20]. For each mechanism, there are several different types of models, which are usually categorized by the existence of traps or recombination-generation centers. The contribution of these different mechanisms largely depends on external operational conditions, such as voltage and temperature.

Timing jitter models have been developed to evaluate the timing performance of SPADs. In SPADs, there is some delay for photon absorptions to successfully trigger output pulses, which means a lag between the detection of a photon and an effective avalanche output. However, the timing performance of SPADs is susceptible to false detection events caused by dark counts. Therefore, the measurement of SPADs’ and SPAD arrays’ temporal response is usually based on a statistical method by measuring a certain number of repetitive output pulses to generate a histogram [21,22,23]. This method is also known as the time-correlated single photon counting (TCSPC) technique. In a typical histogram response of SPADs, the time response of detections varies, which may also be caused by the timing jitter. Accurate timing jitter models can help designers to optimize the timing performance of SPADs so that the fluctuation measured in a histogram can be improved.

Modern SPADs are usually integrated with circuits on one chip to achieve high performance, small size, and reduced fabrication cost. In [24], a comparison was made to compare the cost of different photodetectors. Following the evolution trend of photodetectors, the front-end circuits of SPADs have a significant influence on SPADs’ integration and cost. However, it is difficult to simulate real SPADs with circuits at the schematic level, so designers often face challenges prior to obtaining actual measurements. To address this problem, circuit models using simulation-available components and symbols, such as capacitors, resistors, and MOSFET switches, are proposed to describe the performance of SPADs at the circuit level. Using the circuit models of SPADs, designers can simulate SPADs with quench and reset circuits (QR), readout circuits, and TDCs to gain insight into the overall performance at the system level.

The SPAD physical models and SPAD circuit models have enabled designers to have more freedom to adjust their design for optimized performances prior to fabrication, especially for the SPAD designs using advanced CMOS technologies. In recent years, many models have been proposed for the accurate and comprehensive description of SPAD-based detection systems. Therefore, a discussion of SPAD principles and the recent progress of models can provide a clearer idea and guidance for designers to improve their own models and designs.

**Figure 1:**
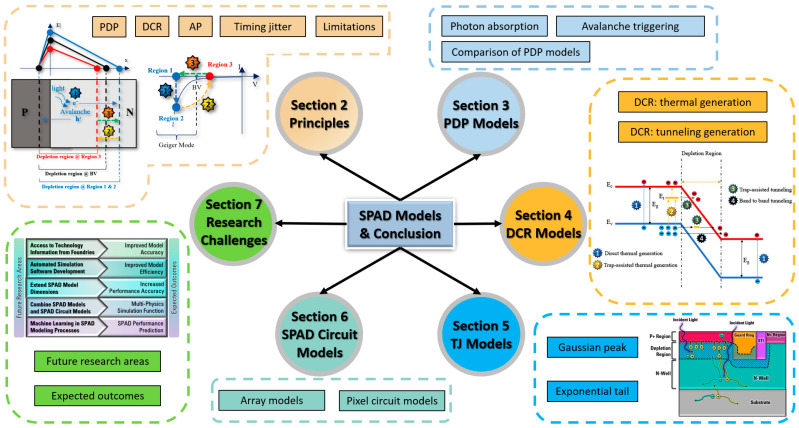
Organization of the SPAD model review paper.

As shown in Figure 1, we will provide a detailed discussion of SPADs’ operational principles, the progress of SPAD models, and circuit models. In Section 2, the fundamental principles of SPADs and their front-end circuits will be introduced. Different PDP models are discussed in Section 3, and DCR models, together with afterpulsing probability models, will be addressed in Section 4. In Section 5, we discuss timing jitter models, and in Section 6, SPAD circuit models are introduced to show how they can help simulate different SPAD parameters at the circuit level. Research challenges and future perspectives will be presented in Section 7. Finally, a summary is concluded in Section 8.

## 2. SPAD Operational Principles

**Figure 2:**
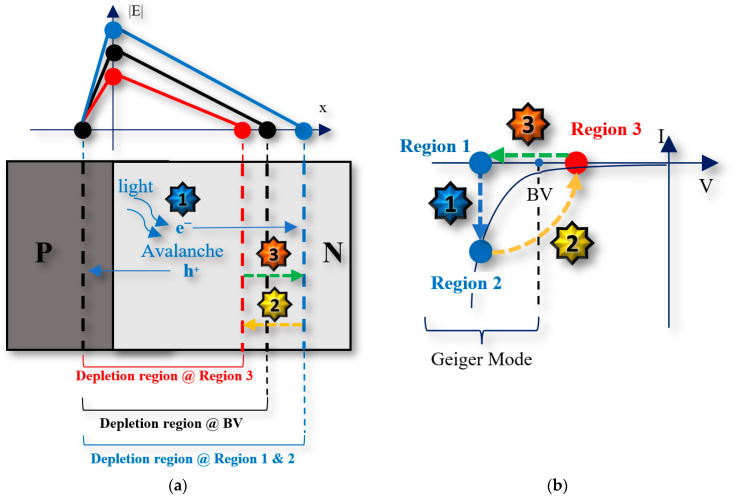
(**a**) Simple SPAD diagram (**b**) Current-voltage (I-V) characteristics.

SPADs are reverse biased above their breakdown voltages [25], and this is commonly known as operating in the Geiger mode. In the Geiger mode, the avalanche current increases rapidly with increased reverse biasing. Therefore, the effective output of a typical SPAD is like a digital pulse, which means that we do not need an analog-to-digital converter (ADC), thus reducing the cost. A simple SPAD structure and its current-voltage (I-V) characteristics are shown in Figure 2. Ideally, SPADs will be biased above the breakdown voltage. The difference between this bias voltage and the breakdown voltage is called the excess voltage. When there is no carrier, there will be no avalanching current, and SPADs will stay in Region 1 until there is an incident photon or dark carrier that initiates an avalanche. When the avalanche is triggered, the SPAD’s current will increase rapidly, thus entering Region 2. However, SPADs are not able to stay in this region for a long time due to the heating effect of the huge self-sustained avalanching current, which can burn the device. As a result, SPADs must be quenched properly. The most common way to achieve this is to reduce the reverse voltage below the breakdown voltage to suspend the avalanche. Then, successfully quenched SPADs can enter Region 3, after which they will be reset to Region 1 to be ready for the next detection. Other types of SPADs’ quench and reset circuits will be introduced in Section 2.4. In the following sections, several important parameters that are commonly used to describe a SPAD’s performance are discussed.

### 2.1. Photon Detection Probability (PDP)

Photon detection probability (PDP) is a key parameter that describes the detection probability of optical sensors, including SPADs. There is another parameter called photon detection efficiency (PDE), which is simply the multiplication of the PDP and the fill factor. As shown in Figure 2, incident photons can be absorbed and then trigger avalanches when SPADs are in Geiger mode. However, whether the absorbed photon can successfully trigger avalanche events at specific locations depends on the triggering probability, which can be described as a function of position for simplicity. When the photon-generated carriers move under the influence of an electric field in the depletion region, they gain energy. Carriers with a certain amount of high energy (energized carriers) can scatter with a bound electron in the valence band, transferring a certain energy to the bound electron and exciting it to a free electron, as in [26]. Therefore, a new electron-hole pair is created, and these newly created electron-hole pairs can also gain energy in the high electric field to ionize more bound electrons. This results in self-sustained chains of impact ionizations. Since the reverse voltage is higher than the breakdown voltage, SPADs do not need a very wide depletion region for accelerating carriers. However, intrinsic layers can be used to improve the absorption of photons [27].

According to the physical process of avalanche events described above, PDP can be defined using two terms. The first term is the absorption probability of incident photons, which gives

(1) $$I\left(x\right)=I_{surface}\times e^{\alpha \left(\lambda \right)x}$$

where *I*(*x*) is the light intensity at a distance *x* below the surface when the incident angle of the light is 0 degrees. *I_surface_* is the light intensity at the surface of SPADs, and α is the absorption coefficient, which depends on the wavelength and material. The second term is the triggering probability, which represents the probability of a carrier successfully triggering an avalanche. Increasing the reverse voltage applied across SPADs’ junctions can increase the triggering probability, thus improving detection efficiency.

### 2.2. Dark Count Rate (DCR) and Afterpulsing (AP)

Dark count rate (DCR) is used to describe the dark noise of SPADs in the units of count per second (cps) or Hertz (Hz). When there is no light, the carriers from different mechanisms can become energetic in the high electric field, initiating impact ionizations and generating pulses that are indistinguishable from photon-induced output pulses. In modern CMOS technologies, SPADs’ DCR can have a large range from tens of Hertz to hundreds of kiloHertz [1,18,28,29,30,31,32,33,34,35,36]. The DCR can originate from various mechanisms, including band-to-band thermal generation, trap-assisted thermal generation, trap-assisted tunneling, and band-to-band tunneling. Each of the mechanisms shows distinguishable temperature and field dependences.

There is another type of noise that can be regarded as a special dark count, which is those counts generated by the later release of carriers that were trapped by defect centers during the primary avalanching process. Due to the intrinsic property of these kinds of false counts, the specific term “afterpulsing probability” (AP) is used to describe it. Because the trapped carriers are from the previous avalanching current, the unit is the percentage that shows the proportional relationship with total pulse counts.

### 2.3. Timing Jitter

When measuring the temporal response of SPADs, the recorded distribution of output pulses can indicate a time delay between the photon absorption and the output. When the photon-generated carriers that initiate avalanche events originate from locations inside the depletion region, the time delay is small, following a typical Gaussian distribution. On the other hand, a longer time delay with an exponential tail indicates that the avalanche events are initiated by the carriers diffusing from the neutral regions to the depletion region [1,32].

### 2.4. Front-End Circuit for SPADs

As shown in Figure 2, to achieve a varying bias for a SPAD, front-end circuits are needed for quenching and resetting SPADs so that SPADs can achieve continuous detections without becoming overheated or burnt out. The basic operational principles of SPADs’ front-end circuits include three stages: sensing, quenching, and resetting. When an avalanche occurs, the front-end circuit must sense the current quickly so that the SPADs can be quenched in a timely manner to reduce the number of trapped carriers during the avalanche. However, to simulate these front-end circuits, circuit models of SPADs are necessary. Inaccurate SPAD circuit models used in simulations will provide inaccurate performance indications of SPAD front-end circuits. In [1], different quench and reset configurations were reviewed and compared.

**Figure 3:**
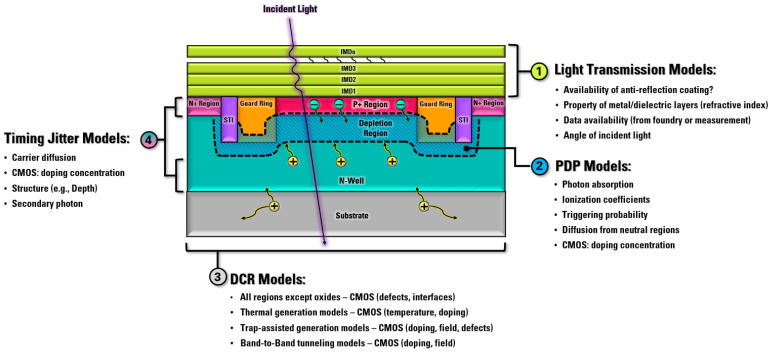
SPAD models graphical diagram with process-related parameters.

Based on the actual physical process of these important parameters of SPADs, researchers are focusing on the development of accurate SPAD models. These models are based on various technology-computer-aided design (TCAD) tools such as Sentaurus and Silvaco [37,38]. A comprehensive review of these models can help to identify future improvements that can be made. Different from many basic analytical models, SPAD models and SPAD circuit models must consider the actual CMOS technology-related parameters, such as doping concentrations, traps’ population, and defects level, as shown in Figure 3.

### 2.5. Limitations

For CMOS-based SPADs, the junction depth is usually determined by specific technologies. In this case, it is challenging to optimize the responsivity of SPADs at a specific wavelength. Customized layers can be implemented to increase detection efficiency at longer wavelengths [16,39]. For other CMOS technologies, the only option is to design multiple junction SPADs. For example, in [40], a dual-junction SPAD was designed in a low-voltage 130 nm CMOS technology, achieving a peak PDP at around 500 nm and 650 nm for the upper and lower junctions, respectively. A triple junction SPAD was fabricated in [41]. However, more efforts need to be put into the improvement of PDP. In addition, SPADs are easily affected by dark noise, as discussed in Section 2.2. In LiDAR applications, SPADs are affected by the background light illumination due to their extremely high sensitivity. As a result, additional circuits need to be added to suppress the noise [4,7,42]. Understanding the mechanisms of important SPAD parameters can help to solve some of these limitations.

## 3. PDP Models

PDP models are developed to simulate the accurate detection probability of SPADs. It is challenging to predict the PDP accurately because fabricated SPADs may suffer from worsening PDP due to technology-related issues such as the depletion region width, doping concentration, and defects [9]. Therefore, the modeling of PDP should be based on physical processes and take technology-specific parameters into consideration. From previous discussions of PDP definitions in Section 2.1, PDP models are also divided into two parts: photon absorption and avalanche triggering.

### 3.1. Photon Absorption

Photon absorption refers to how many photons are absorbed in certain regions. For a given incident photon, its energy determines whether it can successfully excite an electron from the valence band to the conduction band. Without enough energy, the photon will pass through the “transparent” material to such photons. Before the light passes through SPADs and gets absorbed, a certain portion of light has already been reflected at the interfaces of different layers with different refractive indices. However, diverse modern CMOS technologies may have different layers above the active region of SPADs, and these layers may have different optical properties. Therefore, a general model encompassing all technologies and structures is extremely difficult since some technology-related details are not provided. However, a good modeling method can provide designers with clear instructions to build their own models, either by adjusting certain parameters or by considering special processes such as anti-reflection layers [43,44,45]. Many PDP models only focus on the absorption aspect after the complex transmission through different metal and/or dielectric layers above SPADs [46,47]. In such cases, the absorption of photons follows an exponentially decaying distribution away from the surface, as described earlier in Equation (1). For an ideal diode without dark carriers, no avalanche will occur when kept in the dark, even if the reverse bias is above the breakdown voltage. Therefore, photon-generated carriers are regarded as “seed carriers” since they are the initial parts of avalanche chains [20,48,49]. Due to the different bandgaps of various semiconductor materials, there is an upper wavelength limit for each kind of material. However, different applications of SPADs may focus on different wavelength windows. For a typical application of SPADs in PET, the wavelength windows are usually below 500 nm, but this depends on the scintillators used [50]. To meet the requirements for different applications, different junction depths can be used to have different wavelength responses. In some cases, multiple-junction SPADs are designed to achieve detection for different applications [40,41,51].

As shown in Equation (1), simple light absorption is commonly used in many PDP models. Some researchers have attempted the inclusion of light reflection in the PDP models in order to be closer to real performances [44,45]. Light with different incident angles has different reflective properties, which has resulted in several challenges in developing accurate light transmission models. Monte Carlo simulation can be used to simulate carriers randomly. However, this method can be time-consuming and unreliable due to the number of simulated carriers being only a small number compared to total carriers in real conditions [46].

Regarding the light transmission part of PDP models, if the light reflection is not considered, there will be a difference between simulated results and measurements. Simulated PDP versus wavelength curves are smooth, while measured curves usually have many ripples. These ripples are believed to be caused by reflections between different layers, such as dielectric and metal layers [52]. There are two methods to make model-simulated results closer to real measurements: one is to modify SPADs’ structures to reduce the reflection, and the other way is to introduce a full simulation of light transmission.

For modifying the SPAD’s structure, it is possible to add an anti-reflection layer (ARC) above the active region in some technologies. A SPAD fabricated using 350 nm modular high-voltage CMOS technology has been designed with an ARC layer above the active region [44]. With the implementation of ARC layers, the light reflection can be largely reduced at specific wavelengths, and the ripples of the PDP response can become very small. Regarding full light absorption, challenges exist due to certain parameters not being available to designers. In [52], a comprehensive light transmission model that considers passivation layers was simulated using CST Microwave Studio. By doing so, simulated results also show a PDP response with many ripples, which are close to real measurements.

### 3.2. Avalanche Triggering Probability

**Figure 4:**
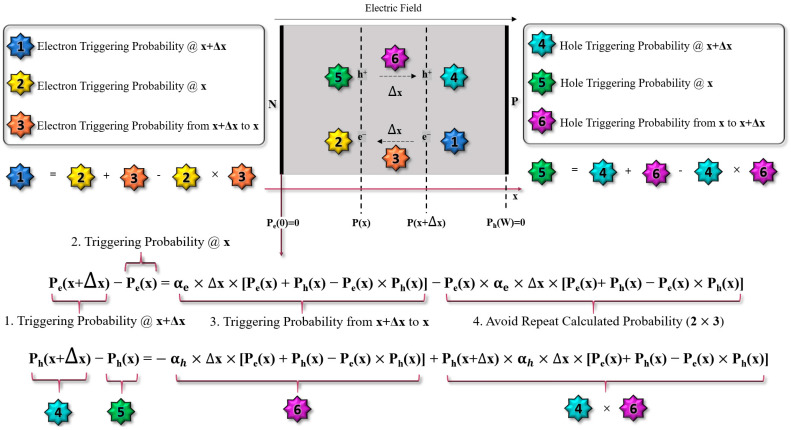
Triggering probability diagram.

The avalanche-triggering probability models focus on the possibility of a carrier successfully initiating an avalanche event. As discussed in Section 2, a carrier must obtain enough energy to trigger impact ionization events. Therefore, there is no physical equation rigorously describing the triggering probability. Considering the requirements of both accuracy and simplicity for the triggering probability model, two differential equations were proposed to generally represent the triggering probability. In a simple 1-D depletion region of SPAD shown in Figure 4, the triggering probability of electrons and holes at specific locations can be represented using the terms “*P_e_*” and “*P_h_*”. The triggering probability of electrons and holes can be represented as a function of ionization coefficients and position. When Δ*x* is small enough, the two equations included in Figure 4 can be transformed into a set of differential equations. After the transformation, a more commonly used form of this set of differential equations is expressed by Equation (2), with two boundary conditions. Note that Equation (2) is for the electric field direction shown in Figure 4.

(2) $$\begin{array}{c} {dP}_{e}/dx=-\left(1-P_{e}\right)*\alpha_{e}*[P_{e}+P_{h}-P_{e}P_{h}],\\ {dP}_{h}/dx=\left(1-P_{h}\right)*\alpha_{h}*[P_{e}+P_{h}-P_{e}P_{h}],\\ P_{e}\left(0\right)=0,\;P_{h}\left(W\right)=0. \end{array}$$

Here, *P_e_* and *P_h_* are the avalanches triggering the probability of electrons and holes, respectively. *α_e_* and *α_h_* are ionization coefficients of electrons and holes, respectively. Ionization coefficients are the only parameters that can be adjusted in these equations.

There are many models for ionization coefficients. In 1966, McIntyre assumed that the holes’ ionization coefficients are *k* times the electrons’ ionization coefficients [11]. However, some improvements can be made to this. First, the ionization coefficients used by McIntyre are not used for PDP modeling but rather used for calculating noise spectral density where a linear relation derived from the current multiplication theory was assumed. Such a linear relationship is hard to be measured from modern SPADs. Second, there will be an effective output pulse as long as a self-sustained avalanche occurs. So, ionization coefficient models obtained from APD may not be accurate enough at the high electric field in SPADs. There are many other ionization coefficient models developed which are available for simulation under high electric fields. Recent PDP models based on commercial simulators have adopted different ionization coefficient models according to their SPADs’ structures and applications, and they are shown in Table 1. It should be noted that terms used in different models have varying meanings, as shown in the last column of Table 1.

#### 3.2.1. Van Overstraeten–de Man Model

The Van Overstraeten–de Man Model is based on the Chynoweth law [37,53]. There are two different sets of coefficients used in the equations. One set is for a low electric field, and another set is for a high electric field. The parameters were obtained from the measurements [53]. This ionization coefficient can be used when the electric field is between 1.75 × 10^5^ V/cm and 6 × 10^5^ V/cm.

#### 3.2.2. Okuto–Crowell Model

Different from the Van Overstraeten–de Man model, the Okuto–Crowell model is an empirical model, which means that the model takes input and output data from experiments to find the best fit [54]. In this model, a detailed physical process is regarded as a “black box”. Therefore, there are many fitting parameters for this ionization coefficient model compared to others. The effective electric field of this model is between 10^5^ V/cm and 10^6^ V/cm, based on default values provided in [37]. However, users can adjust the parameters to fit the specifications of their own measurements.

#### 3.2.3. Lackner Model

The Lackner model has a similar form as the Van Overstraeten–de Man model since they are both based on Chynoweth’s law [55,56]. The difference between these models is that the Lackner model has introduced the electric field dependence in the coefficient part of the exponential equation. As shown in Table 1. the model is effective when the electric field is between 10^5^ V/cm and 10^6^ V/cm, a large electric field range compared to the Van Overstraeten-de Man Model.

#### 3.2.4. Bologna Model

This model is more suitable for simulating the junctions when the reverse electric field is small [57]. The highest electric field of the model is 6 × 10^5^ V/cm, the same as Van Overstraeten–de Man model, but the minimum electric field of this model is 5 × 10^4^ V/cm. Such a value is relatively small in the SPAD’s working region since SPADs are usually biased above the breakdown voltage. This model is suitable when simulating SPADs with a wide depletion region (which has a smaller electric field). Another advantage of this model is the wide applicable temperature range, making it suitable for simulating devices with large currents caused by the heating effect.

#### 3.2.5. Local Model vs. Non-Local Model

These ionization models can be applied to simulating avalanche triggering probability, as shown in Equation (2), depending on the SPAD structure, electric field, and temperature. The parameters in the equations used are all related to specific positions. Since the ionization coefficients are related to the electric field, the electric field is a function of position. Therefore, they can be called “local models”. However, a generated carrier must travel a certain distance to gain enough energy to have an ionization collision. This distance is usually called “dead space” since impact ionization cannot occur inside this region, regardless of the electric field [58,59]. To address this problem, some non-local models have been developed [60].

Non-local models become important due to the shrinking of the depletion region’s width with the advancement of silicon technology nodes. Most of the developed non-local models are based on the multiplication theory of APDs, which have intrinsic layers (multiplication layers). In traditional local models, the ionization coefficients are high when the electric field is high, regardless of whether the carrier is newly generated or has traveled a long distance. In non-local models, ionization coefficients are dependent on both the electric field and the distance of being accelerated. For a certain electric field, *E_c_*, a carrier must be accelerated by the field for a distance of *d_c_* to acquire enough energy to trigger an impact ionization event. Okuto and Crowell proposed their idea of non-local property as early as 1974 [61]. Hayat et al. continued to develop the non-local model by using recurrence equations, which take all carriers into consideration [62]. They also extended their work to determine a more accurate distribution of avalanche events [63]. They split the whole multiplication region into several small segments and applied an iterative method to solve the equations. However, this results in added complexity to the PDP modeling process compared to local models.

In addition to the dead space effect, which reduces the gain of SPADs, there is another physical process called photon scattering, which decreases carriers’ energy, thus further reducing the gain or avalanche-triggering probability. Williams and Ramirez have discussed the photon scattering effect of APDs in [63,64]. According to their theory, the photon scattering effect can reset or “cool down” carriers, thus creating an additional dead space as these carriers must be accelerated again. However, it will be much more complicated to fit the experimental data as more features are included. In the silicon-based SPAD area, the photon scattering and the dead space effect are less important than the GaAs-based or InGaAs-based devices, likely due to their higher ionization coefficients, according to [65].

Additionally, as SPADs are operated in Geiger mode, the current gain will not have a linear relationship with the reverse voltage like APDs. As a result, when considering the triggering models for SPAD-based sensors, researchers are more interested in if photons can induce an avalanche rather than the detailed avalanche current value. Another challenge of implementing a non-local model in SPADs is the rapid change of the electric field in the depletion region. In an APD’s multiplication region, the electric field is used to calculate the coefficients, while the dead space is usually assumed to be constant. However, the rapid change of the electric field along the active region has increased the difficulty of determining an accurate dead space since its dead space also depends on its position. In general, there is always a tradeoff between accuracy and simplicity.

Pseudo-local models have the same form as local ionization coefficient models but with calibrated parameters obtained from experiments. One possible solution is to use pseudo-local models to include the dead space effect. The data extracted from the experiment has already considered the dead space effect. Cheong proposed a possible way to link the experimentally obtained coefficients with the non-local ionization coefficients models [66]. Considering this, the local model of ionization coefficients appears to be more convenient and reasonable for SPADs compared to the models used in similar APDs.

### 3.3. Comparison and Discussion of PDP Models

With the development of photon absorption simulations and avalanching triggering probability models, there are many recently proposed PDP models of SPADs that are summarized in Table 2. Regarding photon absorption, some models simply followed the absorption law, which mainly depends on the absorption coefficient, depth, and thickness of the SPAD depletion region [1,26]. In this case, the wavelength dependence of the PDP will certainly be a smooth curve with different peak values. The different positions of the peak indicate the depth of the junction. For example, in [27], a deep and thick depletion region was adopted to achieve enhanced PDP at a longer wavelength. These types of PDP models are good for indicating the general detection efficiency in the whole wavelength range. However, when it comes to the measured data, there are always some ripples in the PDP response of SPADs fabricated using different technologies [19,44,45,47,52,67,68,69,70,71,72,73,74,75]. These ripples are related to the complex transmission between dielectric and passivation layers above the active region of SPADs [45,52,76].

If researchers are interested in the detailed spectral distribution of the PDP over the whole range, then they must pay attention to the modeling of the transmission through the different layers. If the parameters and properties of these layers above the active region are known to designers, then they may simply introduce Monte-Carlo simulation (MCS) to determine the light transmission. However, it is more likely that researchers may not have access to the detailed specifications of the technologies they are using, which means that the thickness and the property of the materials used in the fabrication are confidential. Under these conditions, the accurate modeling of light transmission will be very difficult.

Some specific technologies have the option of using ARC layers to reduce light reflection. For example, the light reflection was reduced to 0.1% after a single-layer ARC was applied when the wavelength was 504 nm, and no ripples were observed in the measured data [43]. In [44], the researchers modeled the PDP with and without the ARC layer and also tried to find the PDP dependence on the thickness of the ARC layer at different wavelengths. Moreover, in the CMOS imaging sensor (CIS) process, the optimization of stacking passivation and dielectric layers is available, thus increasing the total detection efficiency [72,77].

**Figure 5:**
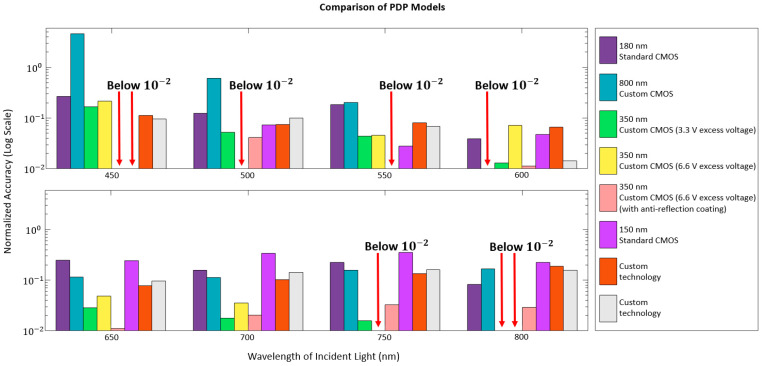
PDP model comparison: (top) Short wavelengths (bottom) Long wavelengths. From top to bottom: Ref [47], Ref [43], Ref [45], Ref [44], Ref [67], Ref [27], and Ref [46].

In Figure 5, different PDP models are compared on a log scale. For each model, the difference between the simulated value and measurements is reproduced from the published data. Larger differences in the short wavelength range are more likely to be observed than those in the longer wavelength range for several reasons. The first reason is that the defects introduced to the device during fabrication are more likely to be closer to the surface, which is the major region for absorbing short-wavelength light. These defects may serve as recombination centers to lower the number of free carriers, thus decreasing the measured detection probability at short wavelengths.

The second reason may be the influence induced by the incident angle. For most small SPADs with diameters of tens or hundreds of micrometers, the incident angle of the long-wavelength light can be simply regarded as 0 degrees. However, with the decrease of the wavelength, the transmission of the light may substantially change as the incident angle changes, thus decreasing the total PDP. This PDP dependence on the light incident angle has also been discussed in [44]. However, the angle dependence of PDP only helps to improve accuracy when researchers are clear about the property of ARC layers or stacked passivation layers. Otherwise, it may introduce more unknown parameters and affect the accuracy of the model.

The size of SPADs also plays an important role in PDP models as SPADs need guard rings to avoid premature edge breakdown (PEB), especially when the doping concentration is increasingly higher in advanced CMOS technologies [29,47,78,79]. With the guard rings around the active region, the doping concentration near the edge of the junction may decrease to a much smaller value. The lowered doping concentration can both decrease the magnitude and bend the direction of the electric field. This has brought two practical problems for PDP models, including the negligibility of this “edge effect” and the detailed distribution of the triggering probability near the edge region.

Most of the listed PDP models in Table 2 did not consider the edge effect. Some PDP models claim that the edge effect is small compared to the large central active region so that the problem can be simplified to a 1-D problem. Some other models simply neglected the edge effect due to the 1-D differential equation used to calculate the triggering probability. However, the edge region will undoubtedly have more effect on the total detection efficiency as the sizes of SPADs are scaling down in modern CMOS technologies. In this case, the conventional 1-D model may not be accurate enough to predict the total PDP, and the PDP is usually overestimated. To solve the accuracy issue of the conventional 1-D PDP models, Liu C et al. proposed a 2-D PDP model to investigate the edge effect by calculating the 1-D differential equations following the actual direction of the local electric field [13]. In this way, the total PDP is the summation of each PDP distributed through the whole active region, with the area as the weighting factor. A similar simulation for finding the SPAD’s edge effect was conducted in [80].

The decreased PDP caused by the edge effect is also believed to be more dominant in the short wavelength range because the junction area is close to the surface. For longer wavelengths, the diffusion of the carriers from the neutral region may have a greater contribution, thus having the highest triggering probability at the boundary. Under this condition, the edge effect is more significant in short wavelengths for shallow SPADs. Therefore, the dependence of the edge effect on the wavelengths deserves further investigation.

In Table 2, most PDP models, including newly proposed models, are still based on the local or pseudo-local model due to their simplicity and reasonable accuracy. For each of the listed models, the average accuracy at the peak is digitized according to the published data. Note that the calculation is done between 450 nm to 800 nm with a step of 50 nm for comparison purposes, as shown in Figure 5. Depending on the targeting wavelength range, designers should choose appropriate modeling methods to simulate the performance of PDP.

Based on the above analysis, the detection efficiency can be improved by increasing the photon absorption probability and the excess voltage. Between these two choices, photon absorption has a more complicated physical process, while increasing the excess voltage seems to be an easy solution that directly improves the detection efficiency. Some CMOS SPADs can be biased at more than 10 V above the breakdown voltage [72,81,82]. However, the dark carriers will also increase with the increase of the excess voltage. The increasing triggering probability affects not only the photon-generated carriers but also the dark carriers. In SPADs, electronic noise is less discussed compared to the photomultipliers (PMTs), due to their intrinsic digital property [1]. Instead, when evaluating the noise performance of SPADs, DCR is more commonly used. In this situation, accurate models of DCR serve as the basis for optimizing the PDP.

## 4. DCR Models

As discussed in Section 2, CMOS SPADs’ DCR performances considerably vary, which poses challenges for the design and measurements of SPADs. For example, if a SPAD was fabricated with a large active region using standard CMOS technologies, the DCR measurement may easily saturate the oscilloscope [83]. As a result, photon detection events can no longer be measured. Similar to PDP models, choosing proper models for DCR also should take the physical process into consideration. Some CMOS technologies can achieve very low dark counts, while others can achieve more compact integration with the penalty of much higher dark counts. For a 0.35 µm high-voltage CMOS (HV-CMOS) technology, it can easily achieve over 10 times smaller DCR compared to a 130 nm standard CMOS technology [84]. Therefore, the influence of the CMOS process is significant in DCR modeling. The difference between PDP models and DCR models is that optical property is one of the key characteristics being investigated in PDP models, but the defects and temperature properties are more frequently discussed in DCR models.

**Figure 6:**
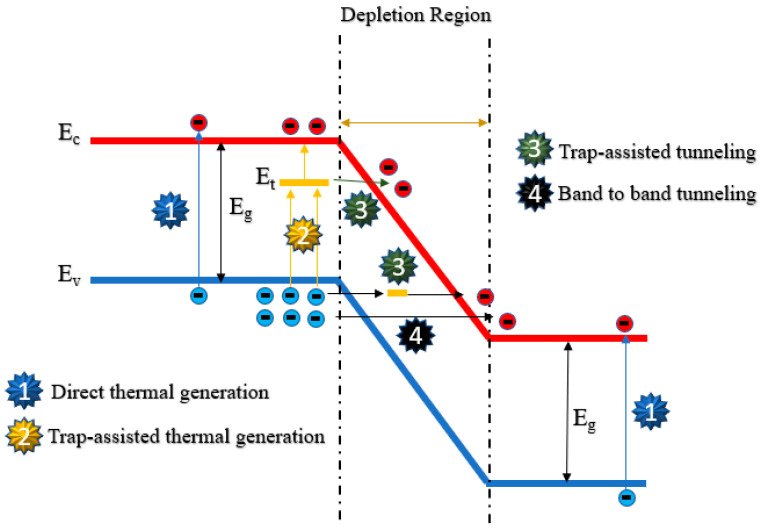
Four major mechanisms of DCR in SPADs.

The force of exciting electrons from the valence band to the conduction band can be any type of carrier other than from the absorption of photons in DCR models. The most challenging work in DCR modeling is to investigate the accurate contribution of different mechanisms shown in Figure 6.

### 4.1. Thermally Generated Dark Counts

One of the basic mechanisms is thermal generation since carriers gain more energy as temperature increases. Therefore, dark counts due to thermal generation usually show a strong dependence on temperature [85]. Note that thermal generation mainly consists of two different mechanisms: direct thermal generation and trap-assisted thermal generation. Direct thermal generation requires more energy due to a large bandgap, thus being negligible in the low-temperature range. Trap-assisted thermal generation, also known as Shockley–Read–Hall (SRH) recombination, accounts for the thermally generated dark carriers in most cases, except in extremely high temperatures. The simple SRH recombination can be represented by Equation (3),

(3) $$R_{SRH}=\frac{np-n_{i}^{2}}{\tau_{p}\left(n+n_{i}\times e^{\frac{E_{t}-E_{i}}{kT}}\right)+\tau_{n}\left(p+n_{i}\times e^{\frac{E_{i}-E_{t}}{kT}}\right)},$$

where the terms n, p, $n_{i}$ are electron densities, hole densities, and intrinsic carrier densities, respectively. $E_{t}$ and $E_{i}$ are the trap energy level and the intrinsic Fermi level, respectively.

From Equation (3), SRH generation has a strong dependence on temperature. In addition to the temperature dependence, electric field dependence is also found in SRH recombination. Hurkx et al. have introduced the electric field dependence enhancement factor, Γ, into traditional SRH generation [86,87]. The factor can be described by Equation (4).

(4) $$\begin{array}{c} \Gamma =2\sqrt{3\pi }\frac{\left|F\right|}{F_{\Gamma }}{exp(\frac{F}{F_{\Gamma }})}^{2}\\ F_{\Gamma }=\frac{\sqrt{24m^{*}\times {\left(kT\right)}^{3}}}{qћ} \end{array}$$

In this equation, *F* is the local electric field, while the term $F_{\Gamma }$ is a parameter that depends on temperatures and effective carrier mass $m^{*}$. Therefore, the introduced field enhancement factor Γ has both temperature and electric field dependence. The combination of SRH and another trap-assisted tunneling can be categorized as trap-assisted generation since they are highly related to the trap population of fabricated SPADs.

Considering assumptions such as the energy level of traps, defect populations, and temperature, the conventional Hurkx model needs to be improved to estimate the DCR of modern CMOS SPADs due to the increased doping concentration and the complicated information of traps. Based on the enhanced SRH model developed by Hurkx et al. [86,87], Kindt modeled the dark counts by assuming the effective carrier mass is 0.25 times free electron mass [15]. However, the difference between the measured results and simulation became more significant at higher excess voltages [15]. Two reasons might contribute to these deviations. One reason is that only the depletion region is taken into consideration, which means the dark carriers diffused from the neutral region were neglected. The other reason is that there is another mechanism called the “tunneling effect” that may become dominant at high voltages.

### 4.2. Tunneling Generated Dark Counts

Band-to-Band tunneling (BTBT) becomes the dominant source of dark carriers when the electric field is larger than 9 × 10^5^ V/cm at room temperature, according to [86]. In such a high electric field, a direct tunneling effect from band to band is more likely to occur. Hurkx also proposed a direct BTBT model to describe this physical phenomenon. The original Hurkx BTBT model can be expressed by Equation (5).

(5) $$R_{BTBT}=-B{\left|F\right|}^{\frac{5}{2}}D\left(F,E,E_{fn},E_{fp}\right)\exp\left(-\frac{F_{o}}{\left|F\right|}\right),$$

where the term *D*(*F*,*E*,*E_fn_*,*E_fp_*) is determined by the bandgap, electric field, and electron and hole Fermi levels. The parameter *B* is determined by fitting experimental results, which are also temperature-dependent [87]. However, it is challenging to get exact information regarding traps, such as trap density and trap energy. Moreover, such information can even vary from different fabrication runs, even with the same technology. The trap information does play an important role in DCR models. To resolve these problems, some researchers set some trap information as fitting parameters.

In [19], trap density, doping concentration, and doping gradient coefficients are all set to fitting parameters used in the SRH and BTBT model developed by Kindt and Hurkx. The convenience of implementing traditional models with user-modified parameters is assisted by TCAD simulation tools. The proposed model in [19] simulated results were compared with the measured DCR of a different device fabricated in different technologies, including 0.15 μm CMOS and 0.13 μm CIS technologies [72,88]. The good predictions associated with these works indicate that this modeling approach can be used in different technologies. For the BTBT component of the DCR model proposed in [19], a larger parameter of B in Equation (5) was chosen. 8 × 10^15^ cm^−3^∙s^−1^ was chosen in lieu of 4 × 10^14^ cm^−3^∙s^−1^ by explaining a faster growth of DCR with high biasing voltage. Note that the power factor of the electric field was the same as the value used in traditional BTBT models [87,89]. The experimental results showed that DCR was highly related to doping concentrations.

A similar idea was studied by Knežević et al. in [14], where the influence of guard ring structures was investigated. With a lower doping concentration of the guard ring, the total DCR of devices was simulated to be much smaller than the device without guard rings or with higher-doped guard rings. In this model [14], several assumptions related to trap energy were made. However, the fitting parameter of trap energy has a different value in the SRH model than in the BTBT model, indicating the influence of other physical parameters. An improvement of their model is that they adopted the process-related simulation tool Sprocess, which is more accurate compared to the simulation based solely on ideal geometric structures. The simulated results were compared to the measurements in [90], which also shows good accuracy. However, the fitted value of trap energy was obtained after assuming a certain doping concentration. This means that different doping concentration assumptions will give different fitted values of trap energy. Due to the lack of information from foundries, the only way for researchers to determine the exact doping concentration is from real measurements. The inaccurate doping information did give rise to unexpected DCR performances, which was proven in [91]. In [91], the doping concentrations used in DCR modeling were from measurements. More importantly, the DCR contribution from upper and bottom neutral regions also needs to be considered. For the BTBT models, default values from the Hurkx model are used. The energy level was regarded as a fitting parameter, but the actual doping concentration was used. Thermal generation was also neglected in the neutral regions. Apart from the analysis of DCR from the neutral and depletion regions, the DCR contributions were also modeled in different regions, from the central uniform region to the guard ring region.

Compared to [14], the DCR model was further developed with additional quantitative analysis of contributions from different regions. DCR generated from guard ring structures is believed to dominate the total DCR of SPADs due to the previous underestimation of its doping concentration. In addition to these impurity-related DCRs, the accuracy of temperature-dependent DCR performance was improved by extracting fitting parameters at different temperatures. Moreover, the influence of some specific fabrication steps has been analyzed. For example, the increased DCR caused by implantation damage has been investigated [92]. Most recent DCR models, which are based on TCAD software, have considered the bandgap narrowing effect, which is caused by the high doping concentration, and carriers’ density of junctions [93]. This effect is activated by default in some popular simulation tools, such as Sentaurus. A summary of recently developed DCR models is shown in Table 3.

**Figure 7:**
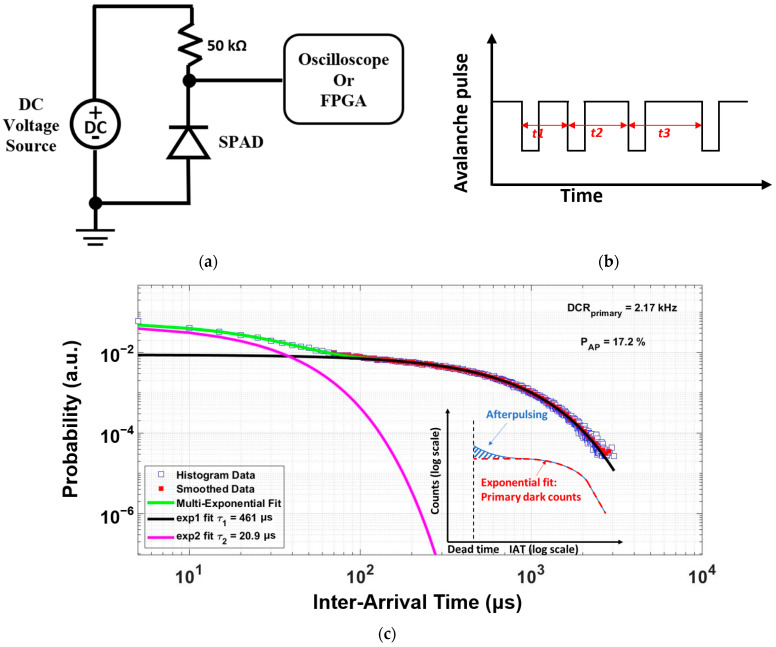
Characterization of SPADs’ timing jitter (**a**) Measurement setup (**b**) Output waveforms (**c**) An example of measured afterpulsing probability with the illustration (bottom right) [29].

With the increased understanding of the physical generation of dark counts, more accurate DCR models based on SRH and BTBT mechanisms are now available. However, when the timing information of SPADs is included, the sources of dark counts are even more complicated. One of the most common sources of this is afterpulsing-induced dark counts. When plenty of carriers are generated due to the avalanche of SPADs, a certain portion of the carriers may be captured by the traps and released after a time delay. If trapped carriers are released during the high voltage or resetting process, they can trigger an avalanche event which is regarded as a false count or a secondary dark count. The release time of trapped carriers is random due to the finite lifetimes of trapping centers [71,94]. The parameter used to describe this phenomenon is AP. The AP is usually measured using the inter-arrival-time (IAT) method, in which the time differences between many consecutive SPADs’ output pulses are recorded to form a histogram. Since AP shows a dependence on the lifetime of traps, the inter-arrival-time (IAT) method is used to find the AP. A simple measurement setup for testing the AP of a PQR-configured SPAD is shown in Figure 7a. As shown in Figure 7b, the time intervals between many consecutive output pulses are measured to form a histogram. Then, the AP can be calculated from the IAT histogram using a multi-exponential fit. Another time delay caused by the diffusion of minority carriers from neutral regions to the multiplication region has a significant influence on the timing resolution of SPADs [1]. This timing resolution is commonly characterized as timing jitter, which is another important performance that needs to be modeled to optimize SPAD design.

## 5. Timing Jitter Models

Timing jitter can be described by the widely used full-width-at-half-maximum (FWHM) of the timing histogram, which reflects the deviation between the detected output and the corresponding incident photons of SPADs. One of the major physical mechanisms that influence this FWHM is the avalanche build-up time. Considering a realistic SPAD circuit and measurement setup, the avalanche current should reach a specific value to have an effective output. However, this time difference from the initial ionization to the output varies, which brings practical considerations to timing jitter models. In [95], the researchers proposed a 2D model to explain how the “spreading of the impact ionization” affects the avalanche current rise time. In their model, there are two mechanisms: the drift and diffusion of the generated free carriers and the carrier-emitted secondary photons. For a typical SPAD with either a p+/N-well junction or an n+/P-well junction, the current rising time reaches the minimum when the initial impact ionization happens at the center, according to [95]. Under such conditions, generated carriers can travel in any direction, causing the largest probability of more impact ionization events in unit time. The second mechanism proposed in [95] is the spread of avalanche events caused by the absorption of secondary photons since the secondary photon created by the emission can be reabsorbed in a random place. According to the hot electron emission theory, the photons emitted by hot carriers are mostly infrared photons, which have wavelengths larger than 780 nm [95,96]. Thus, the timing jitter caused by the reabsorption of secondary photons can be neglected in SPADs with a small size and a thin depletion region.

**Figure 8:**
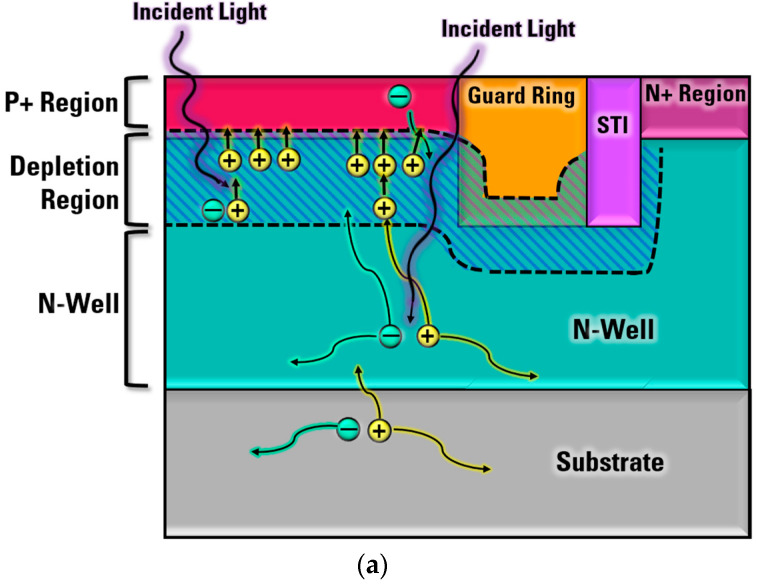

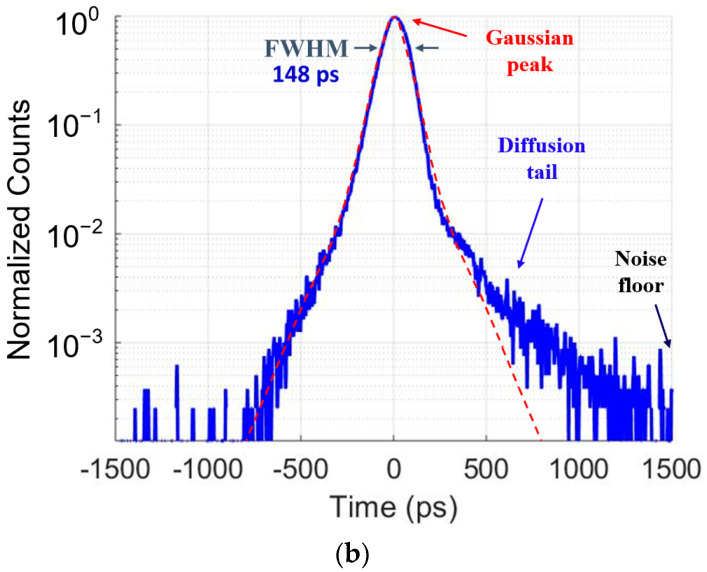
Timing jitter (**a**) Schematic diagram (**b**) An example of measured temporal response [97].

In addition to the carriers generated inside the depletion region, there are minority carriers generated in neutral regions and diffused to the multiplication region. These carriers can act as “seed carriers” to trigger an avalanche pulse. The time delay before traveling into the multiplication region differs depending on the location of photon-generated carriers in the neutral regions. The measurement of timing jitter is usually based on the statistical counting of the output pulses. A typical temporal response of SPAD can have three different components: a Gaussian peak with a small-time delay, an exponential tail with a longer time delay, and random noisy waveforms, as shown in Figure 8a,b [97]. The fast Gaussian peak represents the avalanche event initiated by the carriers in the multiplication region. The exponential decaying tail originated from the diffusion of the carriers. Therefore, the timing jitter in SPADs is quite different from the one defined in other fields, such as oscillators [98]. In this case, many SPAD timing jitter models take the fast Gaussian peak and the exponential tail as two separate components of the total temporal response, as expressed by Equation (6).

(6) $$T_{j}\sim T\left(\mu ,\sigma ,t\right)+P_{neu}\left(t\right),$$

where *T* is a Gaussian function of time with a specific mean value *μ* and deviation *σ*. The second term $P_{neu}$ depends on the width of neutral regions and diffusion lengths (diffusion coefficient) of the minority carriers, as given by Equation (7) from [99]

(7) $$t=\frac{w_{n}^{2}}{\pi D_{c}},$$

where $w_{n}$ is the thickness of the neutral region and $D_{c}$ is the diffusion coefficient of the minority carriers. SPAD-based imaging systems such as LiDAR and PET require timing information. The resolution of the image can be improved by reducing the timing jitter because the detection of photons will be more accurate within a smaller time interval. This interval is also characterized using FWHM, which can vary from tens of picoseconds to hundreds of picoseconds [16,17,28,39,100]. Such a wide range indicates that timing jitter models are generally needed to predict the timing resolution before any fabrication.

The fast Gaussian peak can be modeled by simulating the avalanche build-up time by using the multiplication theory of current. In [101], a threshold avalanche current is set to 100 μA to find the statistical distribution of avalanche build-up time. The exponential tail is based on the simulation of carriers’ densities at both boundaries of the depletion region. In this case, the accuracy of timing jitter models also relies on the ionization coefficients model. If a more accurate distribution of triggering probability is calculated, the timing response will also become more accurate.

## 6. SPAD Circuit Models

Currently, SPADs fabricated in standard CMOS technologies are integrated with other CMOS circuits. Recently, active quenching and reset (AQR) configurations have been widely used due to their fast response, small area, and configurable dead time. The quenching and resetting times have evolved from the microseconds range in passive configuration to several nanoseconds in recent active configurations [28,102,103,104,105,106,107,108,109,110]. To simulate the performance of these advanced front-end circuits, SPAD circuit models are needed. Such circuit models are used to describe the behavior of a real SPAD in integrated circuits, and they are also called SPAD behavior models. The very basic behaviors of SPADs include three stages: (1) below-breakdown (quenched), (2) above-breakdown with no photon, and (3) above-breakdown with incident photons, as described in Section 1. When the voltage across the circuit model is below the breakdown voltage, the circuit is nearly open, and no current flows. When the reverse voltage across the terminals is greater than the breakdown voltage, upon the detection of a photon, there will be a large current due to the avalanching effect.

### 6.1. SPAD Pixel Circuit Models

**Figure 9:**
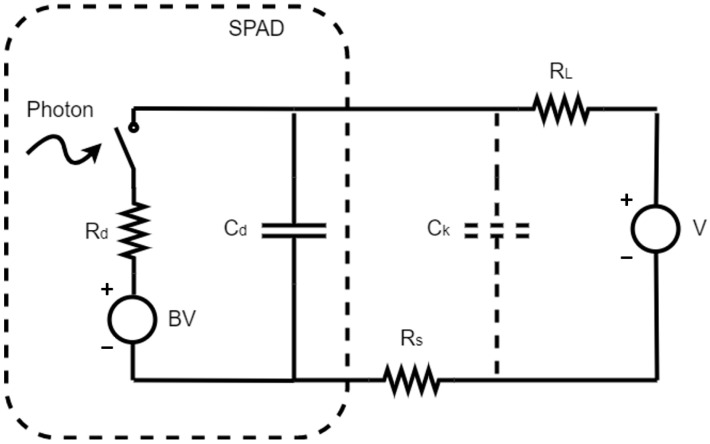
Simple SPAD circuit model (dotted box) with passive quench and reset circuit.

The simplest circuit model is constructed using a voltage source, resistor, and capacitor, as shown in Figure 9. When the voltage is larger than the breakdown voltage, the switch is closed, and the current flowing through the terminals will depend on the avalanche resistance $R_{d}$ and excess voltage V−BV. This simple model can simulate the basic behavior of the SPAD when the timing response is not crucial. Notably, internal avalanche resistors and capacitors affect both the amplitude and timing of avalanche pulses. Inaccurate values of these parameters used for simulation in circuits can lead to a significant difference in measurements. From the measurement of the I-V characteristics of a SPAD, the avalanching current after the breakdown has a nonlinear characteristic [111]. Therefore, the avalanching resistance cannot be regarded as a constant value for accurate modeling. Some researchers improved the circuit model by replacing the constant resistor with piecewise linear resistors [112,113]. This piecewise resistor element varies as the current changes, so it can be implemented using Spice or Verilog-A. Moreover, to improve the accuracy of the amplitude and timing, the circuit model should also be improved to simulate the common performance properties, such as dark count, dark current below breakdown voltage, and temperature effects. In [113,114], the forward region, reverse region, and breakdown region were all included. Specifically, the self-sustained avalanche was simulated by setting a threshold current of 100 µA. When the avalanche current dropped below this threshold, the avalanche was not sustainable and self-quenched. In [99], thermally generated dark counts were introduced to achieve the statistical simulation of avalanche pulses caused by incident photons and dark counts. Zeng et al. further developed the circuit model by introducing the band-to-band mechanism, which is a dominant source of dark counts in nanoscale devices [115]. In this model, there were different triggers for different mechanisms, so they did not interfere with each other but could be simulated at the same time. With the consideration of a piecewise resistor, more accurate capacitance fitted to measurements, and more complete mechanisms, the SPAD circuit model will be more accurate in the circuit simulation. This has enabled the possibility of the system-level simulation of SPADs and their front-end circuits.

### 6.2. SPAD Array Circuit Models and Crosstalk

According to the SPADs’ operational principles, the dead time between detection events can affect the data throughput. To achieve continuous detection and increase the data throughput, SPADs and their readout circuits can be integrated into arrays. In SPAD arrays, all SPADs can be operated at the same time or in different time windows, characterized as the free-running mode and time-gated mode, respectively. When SPADs are integrated in arrays, there are some undesired crosstalk effects. These effects can trigger false counts in SPADs that are near the SPADs which are detecting the primary events. Crosstalk in a SPAD array can be categorized into two types: electrical crosstalk and optical crosstalk, depending on how the interfering signal is transmitted [116].

**Figure 10:**
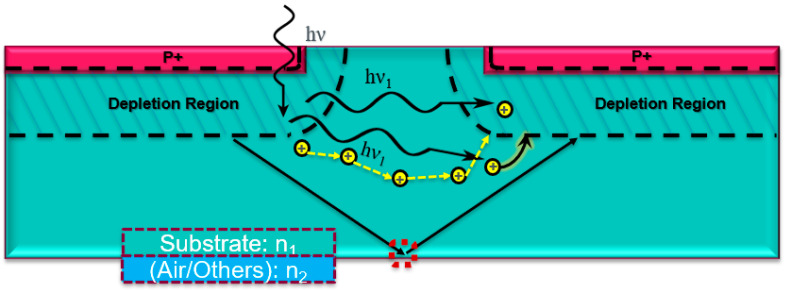
Crosstalk in a SPAD array without isolation (dashed arrow: electrical crosstalk; solid arrow: optical crosstalk).

Mechanisms of SPAD array crosstalk are explained in Figure 10. For electrical crosstalk, some free carriers generated during the primary avalanche after the detection diffuse to the depletion region of neighboring SPADs. These diffused carriers can start initiating avalanching processes. For optical crosstalk, secondary photons are generated due to the electroluminescence effect, which means that photons can be reabsorbed in the depletion region or within one diffusion length of the depletion region of neighboring SPADs. In addition, some secondary photons can be reflected by the bottom layer. The optical properties of the bottom interface may determine how many photons can be reflected. To reduce or prevent crosstalk, there are several possible solutions, such as changing the operation strategy, adding trench isolations, or adding opaque materials between different SPAD pixels.

In a time-gated SPAD array, only a certain portion of SPAD pixels are active for detection in specific time windows, while others are disabled. Under such conditions, the noise or false count caused by crosstalk can be reduced. To further suppress the crosstalk, in a specific time window, SPAD pixels far away from each other can be activated while neighboring pixels are disabled. This is because the crosstalk probability is inversely related to the distance between pixels [117,118]. From the SPAD design perspective, depending on the technology used, shallow trench isolations (STIs) or deep trench isolations (DTIs) can be added to prevent the diffusion of carriers [119]. Another way to alleviate optical crosstalk is to add an opaque material between SPADs. However, this could increase the cost of fabrication, and it may not be applicable to CMOS technologies. There are always trade-offs between fill factor, crosstalk probability, fabrication cost, and complexity, which should be carefully considered by researchers for their own design.

## 7. Research Challenges

**Figure 11:**
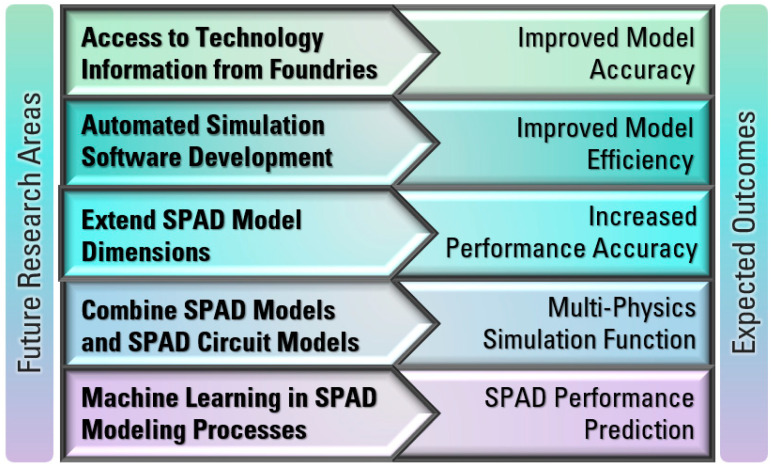
Summary of future research perspectives and potential outcomes.

From the discussions above, improvements in SPAD models and circuit models can help to improve the design and performance of various imaging systems. However, there are still some challenges that need to be addressed to further improve the accuracy and efficiency of modeling SPADs. Current challenges and future research areas are summarized in Figure 11 and further described thereafter.

**Figure 12:**
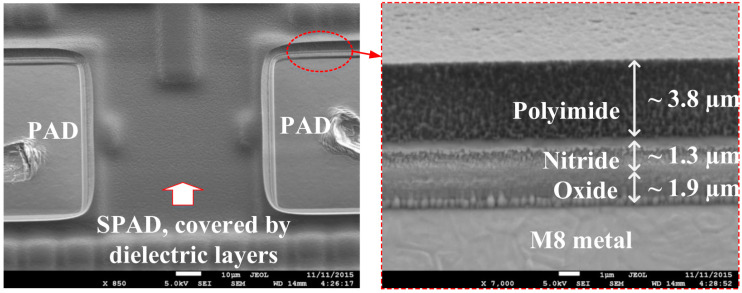
Scanning electron microscope image of SPAD: Top view (**left**) Cross-sectional view of top layers (**right**) [120].

**Figure 13:**
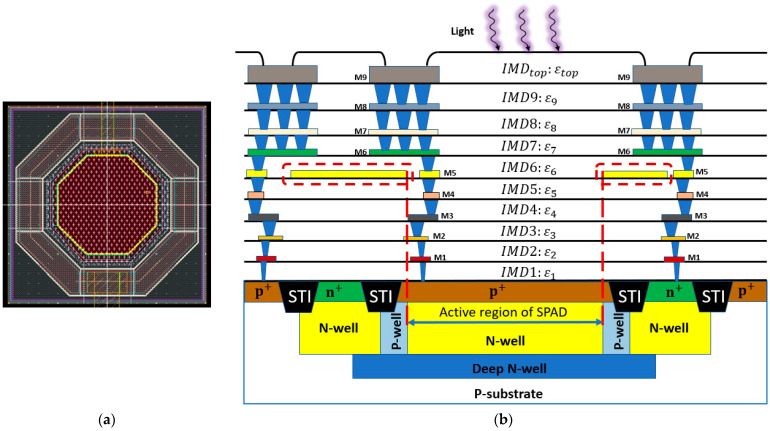
Realistic structure of SPAD based on TSMC 65 nm Standard CMOS technology (**a**) Layout view (top view) (**b**) Cross-sectional view of the design.

- Confidential technology information from foundries has led to difficulties for researchers in developing more accurate models. To have an accurate estimation of certain key parameters in SPAD models, it is important to know the doping profile of each region, the depth and thickness of each region, the material of dielectric layers and defect information, etc. Researchers can extract these parameters directly or indirectly from experimental measurements, but these may be complicated and time-consuming. For example, it may be possible to guess the doping profile by measuring the breakdown voltage. It is also possible to measure the thicknesses of the metal layers and/or passivation layers above the active region from the cross-sectional images using a scanning electron microscope (SEM), thus improving the accuracy of PDP and DCR models. An example is shown in Figure 12, in which the top passivation layers and eighth metal layer M8 are shown with depth information [120].
- However, for a realistic SPAD fabricated using advanced CMOS technologies, it is challenging to get accurate information on each passivation layer. Figure 13a,b shows an illustration of the cross-sectional and layout view of a SPAD design using the TSMC 65 Standard CMOS technology, which has nine metal layers and many inter-metal dielectric layers. The fifth metal layer M5 in the dotted boxes, is used to shield the SPAD, with an opening window to reduce the reflection of the light above the active region. However, there are still many inter-metal-dielectric (IMD) layers with different thicknesses and properties which deserve more investigation for the future improvement of SPAD models.
- When designing a SPAD based on CMOS technology, there are several steps that can be followed to improve the performance. First, avoid crossing metal layers above the active region, which can reduce the light being absorbed in the active region. Second, design a suitable distance between STIs and the active region, usually longer than one diffusion length. This is because that surface generation at the silicon/oxide interface is found to be a major contributor to high DCR [31,34]. Third, the size of the active region should be carefully chosen based on the specific technology. If the size of the active region is too large, the measurement will become a problem since a very high DCR can easily saturate the testing equipment. If the size is too small, fewer photons will be collected in the active region. In addition, the PDP of the individual SPAD may decrease due to the decreased detection efficiency near the guard ring region [13]. A general SPAD model that can include customized parameters would be very useful to device designers.
- The development of simulation tools will also benefit the SPAD modeling process. Currently, TCAD is commonly used for simulating electric parameters of SPADs, including the electric field distributions, ionization coefficients, and breakdown voltage, but not their noise performance [121]. However, these parameters need to be exported to be further processed by other software, such as MATLAB. When there are many parameters to be investigated, the modeling process will be complicated and time-consuming. The development of software automation can significantly improve modeling efficiency.
- The dimensions of SPAD models can be extended from one dimension to two dimensions and even three dimensions. Due to the shrinking of technologies, SPADs can be fabricated in a much smaller size, which should take the edge effect into consideration. However, most of the current SPAD models are in 1 dimension. Some of the models have been developed in 2 dimensions for a better estimation of PDP and DCR [12,13,14]. In 3-dimension models, the performance can be more accurate since they can model these parameters with different SPAD shapes, such as circular SPADs, rectangular SPADs, and octagonal SPADs.
- SPAD circuit models can only be used to simulate the electrical performance of SPADs in integrated circuits. It would be significant progress if SPAD device models and SPAD circuit models could be combined. In this case, the input can be a light signal in the simulation instead of the electric stimuli used in existing SPAD circuit models. However, this method also requires the development of software to include the function of simulating multi-physics in circuits.
- The accuracy of SPAD models also depends on the density of mesh points in a simulation process. Without correct mesh settings, convergence problems may occur. That is to say, it is challenging to adjust the mesh settings in semiconductor device simulation. When it comes to SPAD simulations, the convergence issue can be more important due to the rapid change of physical parameters during the avalanche process. To achieve good accuracy, many calculation iterations are needed, which can lead to additional computational and time costs of modeling. To overcome this problem, machine learning techniques can be adopted in SPAD modeling processes. With machine learning, the design parameters, such as doping concentration, dimension or size, shape, as well as other parameters, can be investigated to see their influence on the specific performance of SPADs. With more data available from simulations, machine learning models can be more accurate. Therefore, predictions of SPAD performances based on different technologies can be made without the need for an iterative modeling process again.

## 8. Conclusions

In this paper, important SPADs’ performance parameters, the progress of SPAD device models, and SPAD circuit models were reviewed to provide a comprehensive view of optimizing SPAD designs. For each type of SPAD model, we have provided a detailed discussion and compared different technologies. SPAD models become more accurate as more regions and mechanisms are considered. We reviewed PDP models, DCR models, TJ models, and crosstalk models. Based on the discussion of the development of SPAD models, we presented several important research challenges in SPAD modeling. We believe that researchers can use this study to propose more accurate SPAD models and innovative modeling methods to further optimize the design of SPAD-based detection systems.

## Figures and Tables

**Table 1:** Commonly Used Ionization Coefficient Models in Sentaurus [37].

Model [Ref]	General Form (Electric Field)	Electric Field Range (10^5^ V/cm)	Investigated Parameters	Key Characteristics
Van Overstraeten Model [53]	$\alpha =\gamma \times a\times exp(-\frac{\gamma b}{E})$	1.75–6	Temp dependence: γ Constants: *a*, *b* Electric field dependence: *E*	Two sets of coefficients Editable coefficients
Okuto–Crowell Model [54]	$\alpha =a(1+c(T-T_{0}))E^{\gamma }exp(-{\left(\frac{b\left(1+d\left(T-T_{0}\right)\right)}{E}\right)}^{\gamma })$	1–10	Constants: *a*, *b*, *c*, *d*, $T_{0,\;}\gamma$ Temp dependence: *T* Electric field dependence: *E*	Empirical Model Editable coefficients Wide electric field range
Lackner Model [56]	$\alpha =\frac{\gamma \times a}{Z}exp(-\frac{\gamma b}{E})$	1–10	Temp dependence: γ, *Z* Electric dependence: *Z*, *E*	Wide electric field range Editable coefficients
Bologna Model [57]	$\alpha =\frac{E}{a+b\times exp(\frac{d}{E+c})}$	0.5–6	Temp dependence: *a*, *b*, *c*, *d* Electric dependence: *E*	Wide temperature range Full-temperature calibration

**Table 2:** PDP Models.

[Ref] Year	Technology Type	Excess Voltage	Incident Light Wavelength Range	Wavelength @ Peak Response *	PDP/PDE Difference ** @ Peak Response	Method	Merits	Key Information	Additional Comments
[47] 2021	180 nm Standard CMOS	15–30% of BV ****	450 nm–900 nm	~500 nm	~12%	TCAD + MATLAB	Use measured doping profile	Passivation layer information from foundry (SiO_2_ and Si_3_N_4_)	Achieved improved accuracy
[43] 2020	800 nm Custom CMOS	1 V	450 nm–900 nm	~500 nm	~38%	TCAD + MATLAB	A 61 nm-ARC layer (SiNX) was applied above the active region.	Process-related parameters are known and used	Fitting parameter free High difference at short wavelengths
		3 V							
		5 V							
[45] 2021	350 nm High-voltage CMOS	3.3 V	450 nm–850 nm	~600 nm	~1.3%	CST Microwave Studio + TCAD	Investigated the effect of low-doped epi layer Light transmission was included	A 80 μm-diameter SPAD	Readout circuits limit the PDP measurement below 2 V excess
[44] 2021	350 nm High-voltage CMOS - With ARC	6.6 V	450 nm–850 nm	~650 nm	~1.1%	CST Microwave Studio + TCAD	A 44 nm-ARC layer (Si_3_N_4_) was applied above the active region.	PDP was improved for short wavelengths	PDP vs. ARC thickness PDP vs. incident angle
[44] 2021	350 nm High-voltage CMOS - Without ARC ***	6.6 V	450 nm–850 nm	~600 nm	~3.2%	CST Microwave Studio + TCAD	Light transmission was included PDP transmission agrees with the measurement	Long wavelength response was enhanced Lower doped epi-layer used	Without anti-reflection layer
[67] 2016	150 nm Standard CMOS	3 V	350 nm–800 nm	450 nm	~0%	TCAD	Compared to [33]	-	Light transmission was not included
[27] 2010	Custom Technology	5 V	400 nm–1000 nm	550 nm	~68%	TCAD	Indicated the effect of fabrication variations	Okuto–Crowell ionization coefficient model	Limited information on layers above the active region
[46] 2009	Custom Technology	3 V	400 nm–1000 nm	550 nm	~61%	TCAD	Custom technology was used Considered light transmission	Okuto–Crowell ionization coefficient model A 200 μm-diameter SPAD	Limited information on layers above the active region

* Peak response refer to the simulated result; ** |(Measured Data−Simulated Data)/Mesured Data|, data estimated from published sources, with a step of 50 nm-wavelength; *** ARC: anti-reflection coating layer; **** Breakdown voltage from measurements.

**Table 3:** DCR Models.

Ref	SRH *	TAT **	BTBT ***	Tools/Methods	Comments
[87]	Yes	Yes	Yes	Analytical	Assume electrons’ and holes’ lifetimes are equal A mid-gap trap energy was assumed
[86]	Yes	Yes	Yes	Analytical	7 parameters were obtained by fitting measurements with simulations
[15]	-	Yes	-	Analytical	Only depletion region was considered Single trap energy level was assumed
[19]	Yes	Yes	Yes	TCAD ****	Use trap population as fitting parameter Use doping concentration and gradient coefficients as fitting parameters No change to the SRH and BTBT model
[14]	Yes	Yes	Yes	TCAD	Trap energy is assumed to be 0.15 eV higher than the middle bandgap Compared different guard ring structure Assuming a pure Boron process
[91]	Yes	-	Yes	TCAD	Doping and trap information are obtained from measurement Considered the contribution from neutral regions Trap energy level is 0.19 eV for SRH model Fitting parameters are obtained at 125 K and 325 K
[92]	Yes	-	Yes	TCAD	Considered the damage of implantation

* Shockley–Read–Hall recombination; ** Trap-assisted tunneling; *** Band-to-band tunneling; **** Technology-aided computer design.
